# A proximity labeling method for protein–protein interactions on cell membrane†

**DOI:** 10.1039/d1sc06898a

**Published:** 2022-04-30

**Authors:** Qiongyu Li, Yixuan Xie, Rachel Rice, Emanual Maverakis, Carlito B. Lebrilla

**Affiliations:** Department of Chemistry, University of California Davis Davis California USA cblebrilla@ucdavis.edu; Department of Dermatology, School of Medicine, University of California Davis Davis California USA; Department of Biochemistry, University of California Davis Davis California USA

## Abstract

Antibodies targeting specific antigens are widely utilized in biological research to investigate protein interactions or to quantify target antigens. Here, we introduce antigen–antibody proximity labeling (AAPL), a novel method to map the antigen interaction sites as well as interactors of antibody-targeted proteins. As a proof of concept, AAPL was demonstrated using sodium/potassium transporting ATPase (ATP1A1) and epidermal growth factor receptor 2 (ERBB2)-specific antibodies that were modified with an Fe(iii) catalytic probe. Once bound to their target proteins, Fe(iii)-induced catalytic oxidation occurred in proximity of the antigen's epitope. Oxidative proteomic analysis was then used to determine the degree of oxidation, the site of oxidation within the targeted antigen, and the interacting proteins that were in close proximity to the targeted antigen. An AAPL score was generated for each protein yielding the specificity of the oxidation and proximity of the interacting protein to the target antigen. As a final demonstration of its utility, the AAPL approach was applied to map the interactors of liver–intestine-cadherin (CDH17) in colon cancer cells.

## Abstract 2

Modified catalytic antibodies targeting specific antigens are employed to investigate protein interactions and antigen interaction sites.

## Graphical abstract

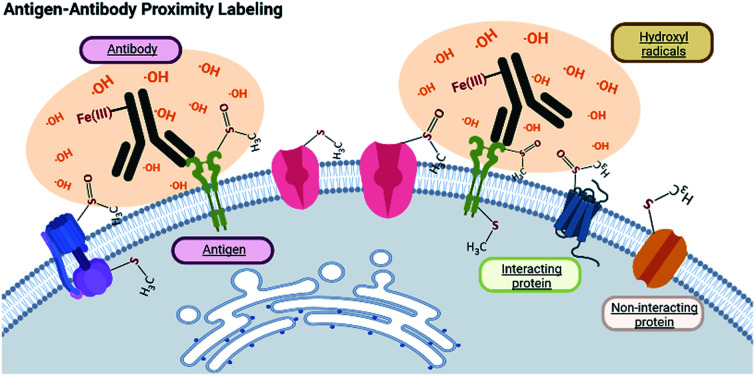

## Introduction

Cell membrane protein interactions are essential in signal transduction and other fundamental biological pathways.^1^ Various approaches have been utilized to study protein interactions. Biochemical engineering approaches such as phage display libraries have been applied to screen the potential antigens of a targeted antibody based on their interactions.^2,3^ With computational tools, potential protein interactions (PPI) can also be simulated based on properties of amino acids and their post-translational modifications.^4,5^ Mass spectrometry (MS)-based approaches, such as affinity purification mass spectrometry (AP-MS) and cross-linking mass spectrometry (XL-MS) have recently emerged for characterizing protein–protein interactions. In AP-MS, enrichment of targeted proteins (baits) is realized through the overexpression of epitope-tagged bait protein, with interactors of the bait being enriched simultaneously. The mass spectrometry analysis enables the identification and quantification of interactors, which reveals the alterations of interaction under different conditions.^6^ This approach has been applied widely to map PPI networks of distinct targeted proteins.^7–9^ However, weak and transient interactions are difficult to be captured by this technique. To capture both strong and weak interactors in XL-MS, interacting amino acids were crosslinked by a linker *in situ*, and the crosslinked peptides were analyzed by high resolution LC-MS/MS.^10,11^ Recent advances in novel linkers as well as in instrumentation to yield higher-order fragmentation, and annotation software allowed XL-MS to be applied widely to mapping protein–protein interactions.^12,13^ In one study, Wheat *et al.* developed a XL-MS platform using a multifunctional cross-linker with advanced separation and MS instrumentation for *in vivo* mapping of PPI thereby generating a comprehensive PPI network *in vivo* in HEK293 cells.^14^

Mass spectrometry-based proximity labeling coupled with proteomic techniques is another widely used approach for probing protein–protein interactions. By fusing the targeted proteins with specific enzymes or catalytic probes, proteins localized near the interaction site are labeled by catalytic reactions that can then be characterized by quantitative proteomics.^15–17^ With proximity labeling proteomics, interactors with both strong or weak interactions of the target are identified. Several studies have employed this approach to characterize protein interactions. Lobingier *et al.*^18^ have developed a proximity labeling method combining with the engineered ascorbic acid peroxidase (APEX) to investigate the interaction network of the protein β adrenergic receptor (B2AR). In another study, a biotinylation reaction was used for the proximity labeling of cell membrane proteins.^19^ However, biotinylation-based approaches require more complicated sample preparation and streptavidin enrichment does not provide information about the site of the interaction. Hydroxyl radical protein footprinting (HRPF) is commonly used for the determination of protein structure and protein–ligand interactions, where the interaction sites are characterized by the site-specific oxidation of proteins and mass spectrometry.^20,21^ Combining proximity labeling and hydroxyl radical protein footprinting, Li and coworkers developed a labeling approach by immobilizing a catalytic Fe(iii) probe on cell membrane sialic acids to map the proteins interacting with sialylated glycans through site-specific oxidation.^22^ The enrichment of interacting proteins was not required with this approach, which assess the extent of the protein–protein interactions by quantifying the degree of oxidation at interaction sites.

In the current study, we developed antigen–antibody proximity labeling (AAPL) as a technique to map the epitopes of a targeted antigen as well as the interactors of the antigen by proximity-dependent oxidation. Two separate approaches were employed to modify the antigen-specific targeting antibody to create a molecule with catalytic function. In one method, the *N*-glycans of the antibodies were modified by changing terminal galactoses (Gal) to *N*-azidoacetylgalactosamines (GalNAz), followed by the conjugation of a synthesized clickable probe, dibenzocyclooctyne-functionalized bromoacetamidobenzyl-EDTA iron(iii) chelate (DBCO-FeBABE) to the GalNAz groups. In another approach, the antibody was directly modified with the iron(iii) chelate of bromoacetamidobenzyl-EDTA (FeBABE) molecule at cysteine residues *via* their free thiol group. Modified antibodies directed against sodium/potassium transporting ATPase (Na^+^/K^+^-ATPase) and human epidermal growth factor receptor 2 (ERBB2) were used as prototypical examples, with the goal of labeling the targeted cell membrane proteins and their interactors in the immortalized prostate epithelium cell line, PNT2, and the human breast cancer cell line, SKBR3, respectively. The oxidations of targeted antigens and their associated proteins were monitored through the quantitative oxidative proteomic analysis to optimize the reaction conditions for specific labeling. The AAPL method with optimized condition was then applied to study the interactors of liver–intestine cadherin (LI-cadherin/CDH17), which is a cadherin-like protein expressed mainly in liver and intestine, as well as the involvement of CDH17 glycosylation playing in its interactome.

## Results

### Determination of optimal oxidation conditions

**Fig. 1:**
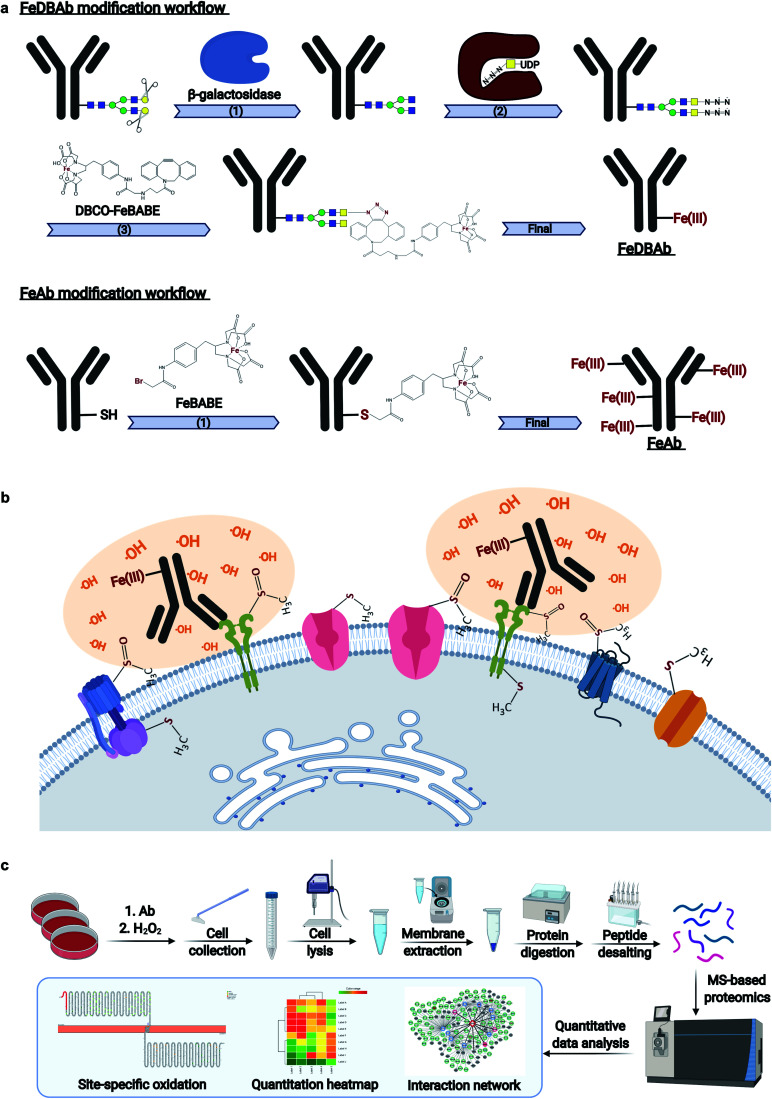
Workflows of AAPL method. (a) Workflows of antibody modification with two approaches including FeDBAb and FeAb. (b) The details of the treatment of cells with modified antibodies and H_2_O_2_. Cells are treated with modified antibodies followed by H_2_O_2_ treatment. Proteins in close proximity to the reaction are specifically oxidized. (c) The workflow of sample preparation, data acquisition, and data analysis.

Monoclonal antibodies specific to human epidermal growth factor receptor 2 (ERBB2) (Herceptin), sodium/potassium transporting ATPase subunit α1 (ATP1A1), and liver–intestine-cadherin (CDH17) were engineered (Fig. 1a) to direct Fe(iii)-induced catalytic oxidation reactions to their target antigens. Specifically, two methods were used. In the first method, antibody Fc region glycans were covalently modified with addition of DBCO-FeBABE (FeDBAb). The second method directly coupled FeBABE to cysteine residues of the antibody's amino acid backbone (FeAb). Each of the resulting antigen-specific oxidizing antibody probes targeted a unique type of cell membrane protein: ERBB2, a cell membrane receptor; Na^+^/K^+^-ATPase, a membrane pump; and LI-cadherin, an adhesin molecule. The modified antibodies were characterized for their glycan modification and binding properties through glycomic analysis and immunofluorescence imaging. The glycomic analysis of modified antibody glycans showed that azido group-containing glycans accounted for around 50% of all types of glycans. Moreover, immunofluorescent imaging revealed that the binding properties of modified antibodies with ether FeDBAb or FeAb were comparable to non-modified antibodies. More details of antibody characterization are included in the ESI.†

Both versions of probes (FeDBAb and FeAb), were tested in parallel using cell lines expressing their respective antigens. After antibody binding, hydrogen peroxide (H_2_O_2_) was added to the culture medium to produce hydroxyl radicals in the proximity of the attached antibody (Fig. 1b). Optimal conditions to minimize overoxidation have been previously reported.^22^ Here, controls to assess background oxidation included cell line only (C1), cell line treated with unmodified antibody (C2), and cell line treated with H_2_O_2_ (C3). For each of the engineered antigen-specific oxidizing probes, treatment conditions were optimized to obtain the optimal oxidation conditions. For FeAb, various ratios of antibody to FeBABE were tested to optimize Fe(iii) conjugation (M1–M3, ESI Table 1†). The resulting oxidizing probes were also assessed under various oxidation conditions to optimize target antigen oxidation. Similarly, FeDBAb optimization also involved varying concentrations of FeDBAb to optimize the oxidation conditions (M4–M6, ESI Table 1†).

**Fig. 2:**
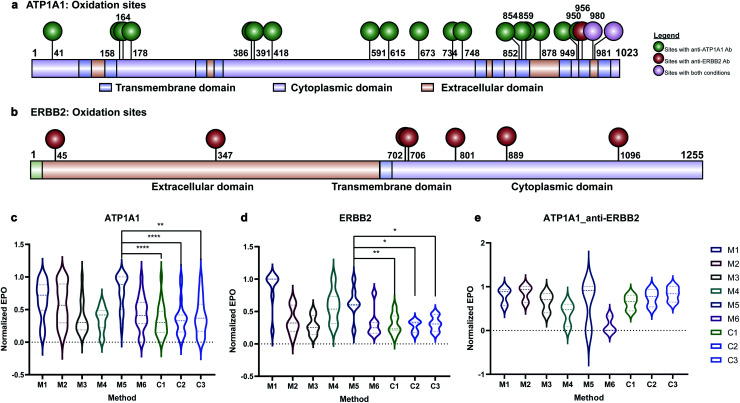
Oxidation of ATP1A1 and ERBB2 with different antibodies. (a and b) Oxidation sites of ATP1A1 with anti-ATP1A1 antibody and anti-ERBB2 antibody are labeled on the domain diagram of ATP1A1. The oxidation sites with anti-ATP1A1 Ab are in green color and those with anti-ERBB2 Ab are in red color. Oxidation sites found in both reactions are in purple color. (c) The EPO of ATP1A1 with anti-ATP1A1 antibody under different reaction and control conditions. The oxidation degree of ATP1A1 under M5 was significantly higher than all three control conditions. (d) The EPO of ERBB2 with anti-ERBB2 antibody under different reaction and control conditions. The EPO of ERBB2 under M5 was significantly higher than all three control conditions. (e) The EPO of ATP1A1 with anti-ERBB2 antibody under different reaction and control conditions. ATP1A1 was not specifically oxidized under any reaction conditions compared to control conditions. All the EPOs in (c–e) are normalized to the condition with the largest EPO.

Membrane proteins were enriched after H_2_O_2_ treatment and subjected to quantitative oxidative proteomics analysis (Fig. 1c). The degree of oxidation for each site on a protein was determined by calculating the ratio of the oxidized peptide intensity to the summed unmodified and modified peptide intensities for each tryptic peptide. Additionally, the extent of protein oxidation (EPO) for each protein was obtained by calculating the ratio of the summed oxidized peptide intensities to the summed total peptide intensities of each protein. The optimal oxidation conditions were determined based on the EPO. The oxidation sites on ATP1A1 and ERBB2 and their positions in the protein domains showed the specificity of each antibody (Fig. 2a and b). It was observed that among conditions M1–M6 and controls, conditions under M5 yielded the highest EPO for both ATP1A1 and ERBB2 (Fig. 2c and d) with their respective antibody.

Antibody cross reactivity was assessed by examining the oxidation of ATP1A1 after treatment with ERBB2-specific oxidizing antibody probe. We noted that three oxidation sites were observed on ATP1A1 after incubation with the ERBB2-specific oxidizing antibody (Fig. 2a); however, none were highly oxidized under M5 conditions (Fig. 2e), demonstrating the high specificity of the AAPL method. To illustrate the EPO of all oxidized proteins, a heatmap was generated for proteins under the M1–M6 and three control conditions (Fig. S3†) using both ATP1A1 and ERBB2-specific oxidizing antibodies. The heatmap demonstrated that similar conditions clustered together, and condition M1 was optimal for FeAb and M5 for FeDBAb.

### Establishment of AAPL interaction criteria for target antigens

Quantitative oxidative proteomic analysis was used to identify proteins in close proximity to the target antigen, which we refer to here as antigen–antibody proximity labeling (AAPL). The antigen together with these co-oxidized proteins can be referred to as an “interaction network”. For the oxidative proteomic analysis, two values were calculated for the total protein oxidation: spectral counts of the oxidized protein and the extent of protein oxidation (EPO), as measured by ion abundances. The combination of spectral counts and ion abundances provided a more consistent method for measuring protein oxidation at the protein-specific level.

A method for calculating topological scores (TopS) of protein interactions in affinity purification mass spectrometry (AP-MS) was adapted here to generate an AAPL score.^23^ In TopS, the spectral counts of each bait-enriched prey protein are used to calculate a TopS score to evaluate the extent of interaction of the prey protein and the bait protein. Similarly, in AAPL the extent of interaction was evaluated based on an AAPL score calculated using the spectral count of each oxidized protein together with its EPO. Detailed equations for the calculations are included in the Method section of the ESI.† The proteins with AAPL scores larger than 0 were considered as more specifically oxidized relative to controls. This method was applied to identify ATP1A1 and ERBB2 interactors, which generated over 40 proteins with AAPL scores pass the 0 threshold under M5 condition.

To generate a more stringent selection criteria for the antigen interactors, we first calculated the average number of oxidation sites for all proteins with AAPL values above 0 and below 1 after ATP1A1- and ERBB2-targeted oxidation. Results differed depending on the targeted antigen. For ATP1A1, a score of 0 corresponded to 2.8 sites, while a score of 1 corresponded to 4.8 oxidation sites. In ERBB2-targeted proximity labeling proteins with AAPL score above 0 had on average 2 oxidation sites, while those with AAPL score above 1 had on average 2.4 oxidation sites. The number of average oxidation sites suggest that proteins with AAPL scores above 1 are more oxidized, indicating a stronger interaction with the target protein.

**Fig. 3:**
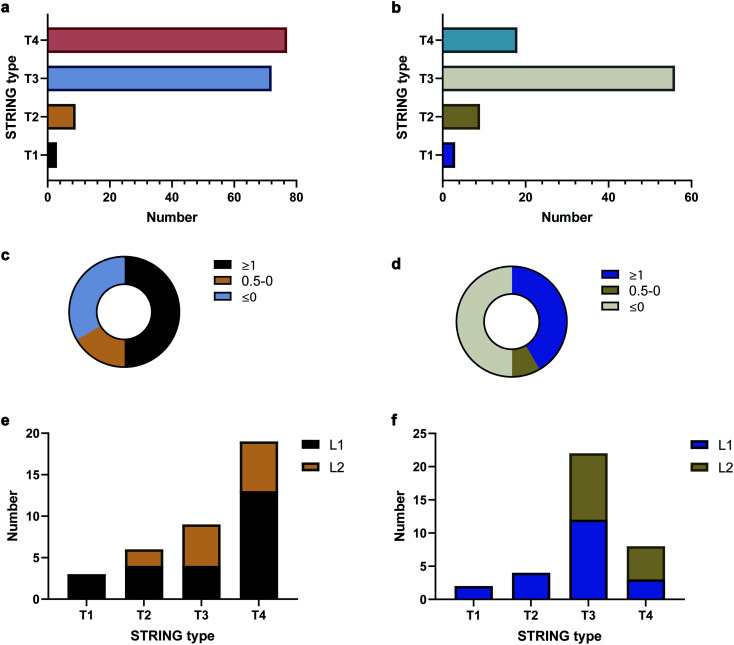
STRING and AAPL classifications as well as AAPL score distributions of oxidized proteins with anti-ATP1A1 Ab and anti-ERBB2 Ab. (a) STRING type distribution of oxidized proteins with anti-ATP1A1 Ab. (b) STRING type distribution of oxidized proteins with anti-ERBB2 Ab. (c) Percentages of T1 and T2 STRING interactors of ATP1A1 with different AAPL scores. (d) Percentages of T1 and T2 STRING interactors of ERBB2 with different AAPL scores. (e) STRING type distribution of AAPL L1 and L2 interactors of ATP1A1. (f). STRING type distribution of AAPL L1 and L2 interactors of ERBB2.

To validate the AAPL scores, the classification of interactors was compared to STRING, which provides scores based on the extent of interaction between proteins using derived literature data. The oxidized proteins were examined with STRING. Oxidized proteins that were known to interact directly with the target protein were classified as Type 1 (T1) and Type 2 (T2) with T1 defined as high confidence interactors, having STRING scores above 0.7, and T2 as medium level interactors, having STRING scores in the range of 0.4–0.7. Secondary interactors, those interacting with T1 and T2 proteins and not directly with ATP1A1 or ERBB2, were classified as T3 interactors. The remaining oxidized proteins were further classified as T4 interactors. The numbers of each type of interactors for ATP1A1 and ERBB2 based on the STRING analysis are summarized in Fig. 3a and b, respectively.

The distributions of AAPL scores for T1 and T2 interactors were then examined. For ATP1A1, more than 50% of the interactors (T1 and T2 combined) had AAPL scores larger than 1. Nearly 15% had AAPL scores in the range of 0–1, and the remainder were below 0 (Fig. 3c). For ERBB2 around 50% of T1 and T2 interactors had AAPL scores >1, with 8% in the range of 0–1, and the remainder below 0 (Fig. 3d). Thus, for the T1 and T2 interactors with AAPL scores larger than 0, a large fraction was with AAPL scores above 1. In addition to AAPL scores, the extent of protein oxidation fold changes (EPO FC) for T1 and T2 interactors was also determined. It was found that for ATP1A1, all the T1 and T2 interactors (corresponding to 9 proteins) with AAPL scores larger than 0 had total EPO FC from 1.5 to 5. For ERBB2, all the T1 and T2 interactors (6 proteins) with AAPL scores larger than 0 had total EPO FC from 1.5 to 3, with one exception. Thus, the STRING and AAPL methods identified similar sets of proteins as strong interactors with the target antigen.

We therefore classified the oxidized proteins based on the AAPL scores with the following criteria. Proteins with AAPL scores above 1 were classified as Level 1 (L1) interactors, while those with AAPL scores in the range of 0–1 and EPO FC above 1.5 were classified as Level 2 (L2) interactors. With this classification criteria, several of STRING classified T3 and T4 oxidized proteins were also found as L1 interactors of ATP1A1 and ERBB2 in PNT2 and SKBR3 cell lines, respectively; while not all the T1 and T2 proteins were primary interactors. We should note interactors identified by the AAPL scores may be dependent on the specific cell lines (or tissue) and culture conditions used in the analysis. In total, four T3 proteins and thirteen T4 proteins were found as AAPL L1 interactors of ATP1A1. Twelve T3 proteins and three T4 proteins were found as AAPL L1 interactors of ERBB2. ATP1A1 and ERBB2 L1 and L2 interactors and corresponding STRING-type associations are summarized in Fig. 3e and f.

### AAPL interaction networks of ATP1A1 and ERBB2

**Fig. 4:**
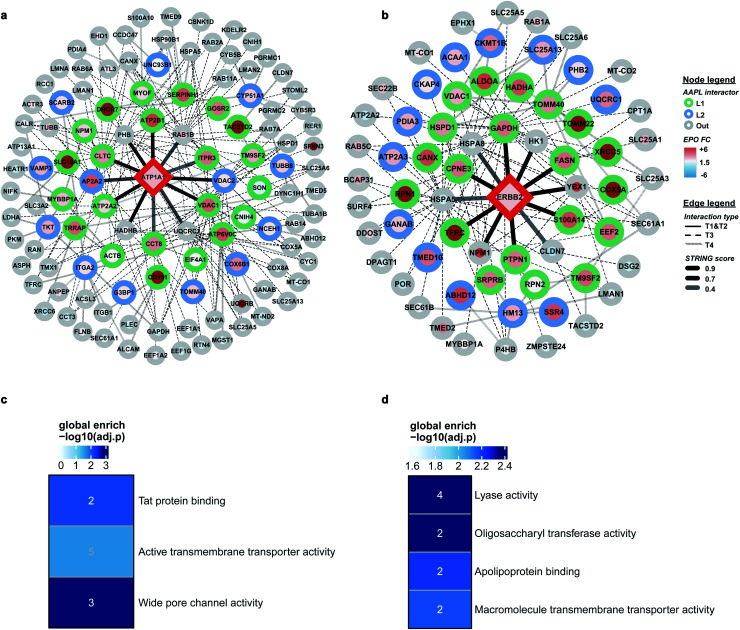
(a) and (b) Interaction maps of ATP1A1 and ERBB2. Interactors are demonstrated as circle nodes with their node frame color represents AAPL interactor types. L1 interactors are in green, L2 interactors are in blue, and oxidized non-AAPL interactors are in grey. The EPO fold change (FC) of each protein is demonstrated as circle filling color. The types of edges represent distinct STRING interactors, with solid lines for T1 and T2 types, dashed lines for T3 types, and backslashes for T4 types. The transparences of edges represent different STRING score ranges. (c and d) Gene ontology enrichment analysis of AAPL L1 and L2 interactors of ATP1A1 and ERBB2.

Comprehensive quantitative networks of AAPL interactors were obtained from STRING (Fig. 4a and b). The network identified both L1 and L2 AAPL interactors of ATP1A1 and ERBB2, with the interaction types represented by different border colors and the EPO scaled with the fill colors. The corresponding STRING types of the interactors are represented by different types of edges. The first order STRING interactions (T1 and T2) are connected with solid edges, interactions of T3 STRING interactors are connected with dashed edges, and interactions of T4 interactors are connected with backward slash lines. The transparency of edges reflects the strength of the interactions, which were evaluated by the combined STRING score. The oxidized peptide and protein results for ATP1A1-AAPL in PNT2 cells and ERBB2-AAPL in SKBR3 cells are summarized in ESI_Tables_I and II,† respectively.

To evaluate how well the AAPL identified interactors of ATP1A1 and ERBB2 were related to the corresponding antigen, gene ontology enrichment analysis was conducted. For ATP1A1 interactors, the analysis of molecular function demonstrated that several genes belong to the groups of proteins that were involved in active transmembrane transporter activity and wide pore channel activity, which were related to the function of ATP1A1.^24,25^ All of enriched molecular function terms had adjusted *p*-value below 0.01, indicating a significant level of enrichment (Fig. 4c).

Interactors of ERBB2 were involved in four molecular functions, including lyase activity, oligosaccharyl transferase activity, apolipoprotein binding, and macromolecule transmembrane transporter activity (Fig. 4d). Among the STRING T1 interactors, G3P (glyceraldehyde-3-phosphate dehydrogenase) and FAS (fatty acid synthase) were identified as AAPL L1 interactors. Four STRING T2 interactors were also identified as AAPL L1 interactors (S100A14, TFRC, PTPN1, and CPNE3). Among these, CPNE3 (copine-3) was demonstrated to interact with ERBB2 through a receptor dependent manner and it may be a novel factor regulating the ERBB2-dependent cancer cell motility.^26^

The specificity of the AAPL method was also investigated by comparing the assigned interactor types of proteins that were found commonly in both ATP1A1 and ERBB2 AAPL networks. A merged interaction network was also generated and is illustrated in Fig. S4.† In total, 5 proteins including GAPDH, TFRC, VDAC1, ATP2A2, and ATP1A1 were found in both interaction networks. It was found that AAPL interactors of ERBB2, including GAPDH and TFRC were not AAPL interactors of ATP1A1, corresponding to their types, they were not increasingly oxidized under ATP1A1 AAPL condition. Similarly, ATP2A2 was a member of the ATP1A1 AAPL interaction network but not the ERBB2 AAPL interaction network. Since ATP2A2 was increasingly oxidized in response to ATP1A1 AAPL while not ERBB2 AAPL, we hypothesize that the degree of protein oxidation is proportional to the degree of its interaction with the target protein. Lastly, VDAC1 was identified as AAPL interactor of both ATP1A1 and ERBB2, corresponding to the observation that the protein was increasingly oxidized under the AAPL condition of both ATP1A1 and ERBB2. These observations demonstrate the specificity of the AAPL method to the targeted antigen and its interactors.

### Application of AAPL to LI-cadherin in Caco-2 cell line

The protein LI-cadherin is a member of the cadherin family found in both liver and intestine. The protein is involved in the morphological organization of liver and intestine.^27^ Although the protein also belongs to the cadherin superfamily that consists of seven cadherin domains, several previous studies illustrated that the LI-cadherin mediated cell aggregation is independent of cytoplasmic interactions. To identify the *in situ* interactors of LI-cadherin, the AAPL method was applied to both undifferentiated and differentiated Caco-2 cell lines. Undifferentiated Caco-2 cells are colorectal adenocarcinoma cells; however, the differentiated cells have properties typical of small intestine enterocytes. It was known that LI-cadherin is highly expressed in intestinal cells.^28,29^ Interestingly, using the AAPL approach, we barely found the oxidation of LI-cadherin in undifferentiated Caco-2 cells, but we found highly oxidized LI-cadherin in the differentiated Caco-2 cells. This is possibly due to the lower expression level of LI-cadherin in the undifferentiated Caco-2 cells leading to less binding of the modified antibodies, which further leads to a lower amount of oxidized proteins that were not enough for oxidation identification. This observation also demonstrated the specificity of the AAPL approach.

**Fig. 5:**
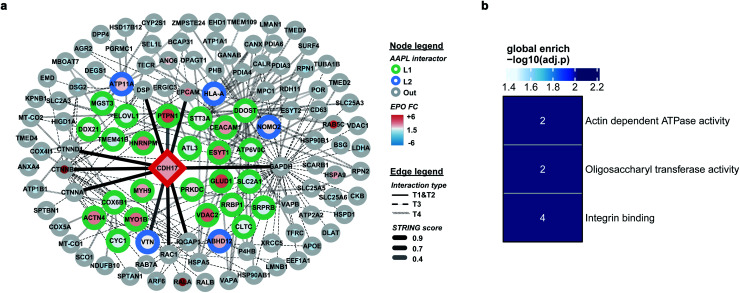
(a) The interaction map of LI-cadherin. Interactors are demonstrated as circle nodes with their node frame color represents AAPL interactor types. L1 interactors are in green, L2 interactors are in blue, and oxidized non-AAPL interactors are in grey. The EPO FC of each protein is demonstrated as circle filling color. The types of edges represent distinct STRING interactors, with solid lines for T1 and T2 types, dashed lines for T3 types, and backslashes for T4 types. The transparences of edges represent different STRING score ranges. (b) Gene ontology enrichment analysis of AAPL L1 and L2 interactors of LI-cadherin.

The employment of AAPL to differentiated Caco-2 cells generated 30 AAPL interactors of LI-cadherin, with 25 L1 interactors and 5 L2 interactors. STRING was used again to organize the oxidized proteins based on known interactions. T1 interactors predicted by STRING, such as catenin subunits and desmoplakin, were found in the cell but were not labeled as L1 or L2 interactors, which is consistent with the independence of the LI-cadherin mediated cell aggregation to the commonly known cadherin-family interacting proteins.^30^ Among the STRING T2 proteins, PTPN1 and VTNC were identified as AAPL interactors, and they have been shown to interact with E-cadherin interaction network.^31,32^ Besides, 12 STRING T3 proteins and 15 STRING T4 proteins were determined as AAPL interactors of LI-cadherin in the differentiated Caco2 cell line. The complete interaction network is shown in Fig. 5a. The gene ontology enrichment result is shown in Fig. 5b. It demonstrates that AAPL interactors of LI-cadherin are involved in several molecular functions including integrin binding. These results demonstrate that AAPL successfully labeled proteins that are known to be related to cadherin. The results for the oxidized peptides and proteins of LI-cadherin in differentiated Caco-2 cells are summarized in ESI_Table_III.†

### Effects of glycosylation on target protein interactions

**Fig. 6:**
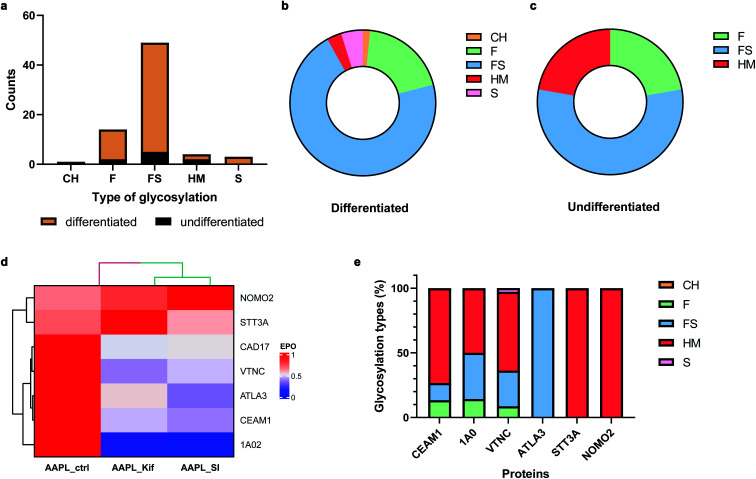
. Effects of glycosylation alteration on LI-cadherin AAPL interactions. (a) Amounts of five types of N-glycosylation of LI-cadherin in undifferentiated (black) and differentiated (mustard) Caco-2 cells. CH, neutral complex and hybrid glycans; F, fucosylated glycans; FS, fucosylsialyl glycans; HM, high-mannose glycans; S, sialylated glycans. (b) and (c) Percentages of five types of N-glycosylation of LI-cadherin in undifferentiated and differentiated Caco-2 cells, respectively. (d) The EPO of LI-cadherin and six glycosylated L1 or L2 AAPL interactors of LI-cadherin in differentiated Caco-2 cells under conditions including optimal AAPL condition (noted as AAPL-ctrl), AAPL-ctrl combined with kifunensine (noted as AAPL-Kif), and AAPL-ctrl combined with 3Fax-feracetylNeu5Ac (noted as AAPL-SI). (e) Glycosylation types of the same six glycosylated L1 or L2 AAPL interactors of LI-cadherin in differentiated Caco-2 cells.

Many cell membrane proteins are glycosylated and often the target of therapeutic drugs. The glycoproteomic analysis of differentiated Caco-2 cells revealed that LI-cadherin in the differentiated cells is generally more glycosylated, compared to LI-cadherin in undifferentiated Caco-2 cells (Fig. 6a). In differentiated Caco-2 cells, LI-cadherin was highly glycosylated with around 70% of sialylfucosylated glycans; the fucosylated glycopeptides of LI-cadherin accounted for 19% of the total glycosylation. Additionally, LI-cadherin contains 3% high-mannose glycopeptides and a small percentage of neutral glycopeptides (Fig. 6b). Compared to differentiated Caco-2 cells, undifferentiated Caco-2 cells contained a higher percentage of high-mannose glycopeptides and less sialylfucosylated glycopeptides, while the fucosylated glycopeptides accounted for similar percentage as in the differentiated Caco-2 cells (Fig. 6c). More detailed site-specific glycosylation maps including numbers of different types of glycans for LI-cadherin in both Caco-2 cell lines are shown in Fig. S5.† The complete glycoproteomic analysis results of differentiated and undifferentiated Caco-2 cells are summarized in ESI_Tables_IV and V.†

Cell surface glycosylation was modified with various inhibitors, including kifunensine (Kif), an α-mannosidase inhibitor,^33^ and 3Fax-feracetylNeu5Ac, a sialyltransferase inhibitor (SI).^34^ These inhibitors are known to dramatically alter cell glycosylation. For example, Kif treatment of Caco-2 cells drastically increases high-mannose *N*-glycan surface expression. In contrast, SI extensively decreased the relative abundances of sialylated glycans. To investigate the effects of glycans on the protein interaction networks, differentiated Caco-2 cells were treated with the different glycosylation inhibitors, followed by incubation with the LI-cadherin-specific oxidizing probe and proximity labeling with H_2_O_2_. The oxidized sequences were then quantified by nanoLC-MS/MS.

In total, there were 30 oxidized proteins that were also glycosylated based on the glycoproteomics analysis. Among these proteins, six of them were identified as interactors of LI-cadherin, with three classified as L1 interactors including CEAM1, ATLA3, and STT3A, and three as L2 interactors including 1A02, VTNC, and NOMO2. To examine whether the extents of the interactions were altered by the inhibitors, we measured the extent of protein oxidation of the native and the glycan modified cells and used the variations to determine extent of interaction. It was found that the oxidation of CEAM1, ATLA3, 1AO2, and VTNC were all decreased when cells were pretreated with kifunensine, suggesting that these proteins interacted less with LI-cadherin. However, there were little to no decrease in the extent of oxidation for STT3A and NOMO2. An identical behavior was observed when the sialic acid expression was decreased by SI (Fig. 6d). Importantly, based on glycoproteomic analysis the former four proteins are all sialylated, while the latter two proteins have primarily high mannose type glycans (Fig. 6e). These results strongly suggested that the interactions of LI-cadherin to the group of four proteins occurred mainly through sialic acid interactions while the two were mainly through high mannose interactions.

## Discussion

Antibody-antigen interactions are generally highly specific, which was leveraged here to map out antigen specific interactors *in situ*. Antibodies labeled with DBCO-FeBABE were found to oxidize their respective protein antigens under optimized experimental conditions. The sites of oxidization on the target proteins were found to be higher in some regions, and it is worth noticing that some of them are located at extracellular regions (Fig. S6†). There was very little cross oxidation between individual protein targets with only background oxidation in the proteins not believed to be targeted by the antibody, even under optimal oxidation conditions. These results suggested that the specificity of the antibody towards the protein can be determined at the site-specific level, with higher oxidation occurring in regions where the antibody binds to the target protein, even when the target protein is in the cell membrane. Although validation of this approach will require additional experimentation, AAPL has promising potential for use in various scenarios. For example, this method has utility as a screening tool. Newly developed antibodies can be easily labeled with DBCO-FeBABE and then be used to probe either cell lines or crude protein extracts, for example, proteins run on gel electrophoresis. Other applications include labeling of autoreactive serum-derived antibodies to identify yet-to-be-described autoantigens.

In addition to the specific oxidation of the target proteins, proteins interacting with the target protein are also oxidized, albeit to a lesser extent. These proteins were primarily those that associated with the target antigen as verified by comparing them to published interactors. The STRING algorithm collates all published interactions and classifies them based on the extent of the interactions. The AAPL approach obtained similar results with proteins identified that matched those obtained by STRING. There were differences between those that were oxidized and those that were previously identified as interactors. The oxidized secondary proteins overlapped with the STRING interactors approximately 50% of the time, when they were present. The major source of the discrepancy could be that protein–protein interactions on the cell membrane may depend on several factors including the specific cell line and culture conditions used. Other proteins were also more highly oxidized than the known STRING interactors, suggesting stronger interactions in the systems studied here than those previously catalogued by STRING. Other factors may also be involved in the oxidation of the secondary proteins including non-specific interactions between the antibody and these proteins. It is worth noting that the interactors characterized through AAPL were in close proximity to the target protein. However, additional methods would be required to further validate the possible direct physical interaction. Nonetheless, AAPL would be well suited for discovering new interactions that can be further validated.

The variations in the oxidation of the AAPL interactors in the cells observed with LI-cadherin presents a new opportunity to elucidate the role of glycosylation in protein–protein interactions. Altering the glycan expression of some AAPL interactors from complex type glycans to high mannose or from sialylated type glycans to non-sialylated glycans affect the extent of oxidation of AAPL interactors, demonstrating the suppressed interactions of proteins to LI-cadherin. Interestingly, LI-cadherin and its AAPL interactors with their extent of interaction affected by altered glycosylation are post-translationally modified by complex type highly sialylated glycans. In contrast, AAPL assessment of cell surface proteins typically modified by high mannose glycans, which contain no sialic acid, yielded similar EPO results before and after Kif or SI treatment. Hence, the AAPL method can be applied to the study of glycosylation mediated protein–protein interactions.

## Conclusions

The antigen–antibody proximity labeling (AAPL) provides a new method for determining the interactors of targeted antigen. The approach for modifying antibodies with DBCO-FeBABE was developed and successfully employed to label the cell membrane antigen and its environment. The reaction conditions of AAPL were optimized for the optimal specific oxidations of targeted cell membrane proteins including ATP1A1 and ERBB2 and their associated proteins in cell lines PNT2 and SKBR3, respectively. With the optimal reaction conditions, the AAPL method enabled the specific oxidation of ATP1A1 and ERBB2 with their corresponding antibodies. The approach also yielded the identification of several known interactors of ATP1A1 and ERBB2, as well as novel potential interactors under the certain cellular environment. Further application of AAPL to the LI-cadherin in both undifferentiated and differentiated Caco-2 cell lines revealed several novel interactors of LI-cadherin. Finally, modifications on cell membrane glycosylation in differentiated Caco-2 cells resulted in decreased oxidation of a subset of AAPL interactors, indicating the essential roles that glycosylation plays in the interaction between these proteins with LI-cadherin.

## Data availability

The mass spectrometry proteomics data have been deposited to the ProteomeXchange Consortium *via* the PRIDE^35^ partner repository with the dataset identifier PXD033466 and 10.6019/PXD033466.

## Author contributions

Q. L. designed and performed experiments, analyzed data, created the figures and wrote the manuscript. X. Y. and R. R. designed and performed experiments. E. M. wrote the manuscript. C. B. L. conceived the idea, supervised the study, and co-wrote the manuscript.

## Conflicts of interest

There are no conflicts to declare.

## Supplementary Material

SC-013-D1SC06898A-s001

SC-013-D1SC06898A-s002

SC-013-D1SC06898A-s003

SC-013-D1SC06898A-s004

SC-013-D1SC06898A-s005

SC-013-D1SC06898A-s006

SC-013-D1SC06898A-s007

SC-013-D1SC06898A-s008
