# Supplementary material for: A proximity labeling method for protein–protein interactions on cell membrane
Source: Chem Sci. 2022 Apr 30;13(20):6028–38. doi: 10.1039/d1sc06898a (PMC9132088; doi:10.1039/d1sc06898a)
Supplement: SC-013-D1SC06898A-s001 [file SC-013-D1SC06898A-s001.pdf]

Electronic Supplementary Material (ESI) for Chemical Science.
This journal is © The Royal Society of Chemistry 2022

A proximity labeling method for protein-protein interactions on cell membrane

SUPPLEMENTARY MATERIAL

Authors

Qiongyu Li[1], Yixuan Xie[1], Rachel Rice[1], Emanual Maverakis[2], Carlito B. Lebrilla[1,3]

Affiliations

[1]Department of Chemistry, University of California Davis, Davis, California, USA.

[2] Department of Dermatology, School of Medicine, University of California Davis, Davis, California, USA.

[3]Department of Biochemistry, University of California Davis, Davis, California, USA.

Correspondence and requests for materials should be addressed to C.B.L. (email: cblebrilla@ucdavis.edu)

Key Words

Proximity labeling proteomics, antigen-antibody binding, protein-protein interaction, glycosylation

METHODS

Cell culture. Three human cell lines including PNT2, SKBR3, and Caco-2 were obtained from American Type Culture Collection (ATCC, Manassas, VA) and studied. All cell media were supplemented with 10% (v/v) fetal bovine serum (FBS), 100 U/mL penicillin and streptomycin (P/S), and non-essential amino acids and 2 mM $_{L}$-glutamine, and were maintained in a humidified incubator at 37 °C with 5% CO_2. Human normal prostate epithelium (immortalized with SV40) PNT2 cells were grown in Roswell Park Memorial Institute (RPMI) 1640 medium, human breast cancer SKBR3 cells were maintained in high-glucose (4.5g/L) Dulbecco's Modified Eagle Medium (DMEM), and human colon carcinoma Caco-2 cells were maintained in Eagle's Minimum Essential Medium (EMEM). All three cell lines were sub-cultured at 80% confluency. To obtained differentiated Caco-2 cells, Caco-2 cells were maintained for another 14 days after reaching full confluency.

Modification of antibodies with DBCO-FeBABE. Antibodies were conjugated to DBCO-FeBABE through their N-glycans. To cleave the terminus galactose, antibodies were incubated with galactosidase at 37 °C for 6-18 h. To further add the GalNAz groups to N-glycans, proteins were incubated with galactosyltransferase and UDP-GalNAz for 18 h at 30 °C. The DMSO solution of DBCO-FeBABE was then added to modified proteins for conjugation and the mixture was incubated at room temperature for 18 h. (**Figure 1a**, FeDBAb modification workflow) After each step, ultrafiltration was conducted to purify modified antibodies.

Modification of antibodies with FeBABE. Antibodies were modified with FeBABE using the method adapted from a previous described protocol.[1,2] For the modification, the antibody was incubated with FeBABE in the conjugation buffer (10 mM MOPS, 200 mM NaCl, 2 mM EDTA, 5% glycerol, pH 8.0) was maintained at 37 °C for 1 h, followed by the ultrafiltration for buffer exchange to storage buffer (10 mM Tris, 0.2 M KCl, 10 mM $MgCl_2$, 0.1 mM EDTA, 1 mM DTT, 50% glycerol, pH 7.6). The modified antibody was maintained at -80 °C until cell treatment. (**Figure 1a**, FeAb

modification workflow). The content of free cysteine residues in antibodies (immunoglobulin gamma 1) which has been reported to be in the range of 0.1-1.1 mole/mole (free SH to IgG)[3] was set as an average of 0.5 mole/mole. Given that the ratio between the FeBABE and SH group is 1 mole/mole, the FeBABE to antibody ratio was sed as 0.5 mole/mole for modification.

Treatment of cells. Cells were kept at 4 °C and treated with modified antibodies overnight at 90% confluency. Then H_2O_2 was added to cells for 30 minutes (min) for oxidation. (**Figure 1b**) To quench the oxidation, the 10 mM Met-NH_2 was used to wash cells. For cell membrane glycosylation modification, 200 mM 3Fax-Peracetyl Neu5Ac or 100 mM kifunensine were administrated to cells for three days. Cells were scraped and spin down to obtain cell pellets for cell membrane extraction and follow-up processes.

Immunofluorescence. Immunofluorescence was employed to validate the binding affinity of modified antibodies. Cells were seeded on glass bottomed FluoroDish™ cell culture dishes (WPI, FL) for imaging. After reaching 60% confluency, cells were fixed with 1 mL of 4% paraformaldehyde (Affymetrix, OH) for 10 min, permeabilized with 1 mL of 0.1% Triton X-100 (Sigma-Aldrich, MO) for 5 min, and blocked for non-specific binding with 0.1 mL of 0.3 M glycine (Sigma-Aldrich, MO). After treatment of cells with modified or original antibodies with Alexa Fluor tags overnight, cells were stained for plasma membrane with the 1000-fold diluted CellMask™ Orange or Deep Red Stain (Thermo Fisher Scientific), and for nuclei with1.6 μM Hoechst 33342 (Thermo Fisher Scientific, MA). Images were taken with the Leica TCS SP8 STED 3X Super-Resolution Confocal Microscope (Wetzlar, Germany) was used for imaging and results were analyzed with ImageJ.

Cell membrane extraction. Cell membrane was extracted with a sequential ultracentrifugation method adapted from an approach developed in another study. In brief, cells were first lysed on a cool rack using a probe sonicator (Qsonica, CT) for 25 seconds. The lysed cells were centrifuged for 10 min at 2,000 × *g* to separate membrane fractions from other fractions

containing nuclear, mitochondria and cellular debris and collect supernatant. After that, the supernatant was ultracentrifuged for 45 min at 200,000 × *g* and 4 °C to pelletize the plasma membrane. The plasma membrane pellet was washed further to remove remaining cytoplasm and endoplasmic reticulum residues with 500 µL of 0.2 M Na_2CO_3 solution and 500 µL of water, through two rounds of ultracentrifugation under same conditions.

Protein digestion. Cell membrane proteins processed further for tryptic digestion. Proteins were first denatured in 8 M urea with sonication for 15 min, followed by reduction with 2 µL of dithiothreitol at 55 °C for 50 min, and alkylation with 4 µL of iodoacetamide (IAA) in the dark and at room temperature for 25 min. After dilution of urea concentration to less than 1 M with ammonium bicarbonate (NH_4HCO_3) solution, 3 ug of trypsin was used for digestion at 37 °C for 18 h. Samples were further desalted with solid-phase extraction (SPE) C18 cartridges and vacuum dried using miVac (SP Scientific, PA) for MS analysis. For glycopeptide analysis, tryptic digested samples were enriched with iSPE®- HILIC cartridges (Nest Group, MA) and also dried with miVac for storage until analysis.

Proteomic and glycoproteomic analysis with LC-MS/MS. The desalted peptides or enriched glycopeptides were reconstituted with water and adjusted peptide concentration to 1 µg/µL using Pierce™ Quantitative Colorimetric Peptide Assay kit. For proteomics or glycoproteomics analysis, around 1 µg of samples were separated with an Acclaim PepMap 100 C18 column (3 µm, 0.075 mm × 250 mm) on an UltiMate WPS-3000RS nanoLC system at the 300 nL/min of flow rate. A 180-min-gradient was used with solvent A containing 0.1% (v/v) formic acid (FA) in water and solvent B containing 90% (v/v) ACN and 0.1% (v/v) FA. Samples were ionized with a Nanospray Flex ion source at positive mode and analyzed with an Orbitrap Fusion Lumos Tribrid Mass spectrometer. The dada-dependent acquisition (DDA) mode was employed, and the parameters are described in another study.[4] In brief, the m/z range of 350-3000 was used for MS1 scan, and precursors ions in the m/z range of 700-2000 with 2-6 charge states were

isolated for MS2 fragmentation. For MS2 fragmentation, fragments were generated with a step-wised high-energy collision dissociation (HCD) and analyzed with orbitrap analyzer.

Label-free quantitative proteomic and glycoproteomic data analysis. For data analysis, Byonic software (Protein Metrics, CA) was used for peptide and protein identification, using the human proteome database downloaded from Uniprot (**Supplementary Table VI**) as the database for searching. The specificity of the *in-silico* digestion was selected at C-Terminals of arginine and lysine for tryptic digestion with the restriction of a maximum of two missed cleavages for digested peptides. For precursors and fragments identification, mass tolerances were set as 10 ppm and 20 ppm, respectively. To eliminate the false positive discoveries, 1% of the false discovery rate (FDR) was used. Several modifications at distinct amino acids were selected globally for peptide identification. Fixed modification carbamidomethylation was assigned at cysteine; variable modifications including the acetylation, deamidation, and methylation were assigned at the protein N-terminus, asparagine and glutamine, and lysine and arginine, respectively. Different types of modifications were selected for glycoproteomics and oxidative proteomics. An in-house developed N-glycan library (**Supplementary Table VII**) was used as glycan modifications which were assigned to asparagines for glycopeptide identification. For oxidized peptide identification, oxidation at 13 common amino acids (M, C, W, Y, F, H, L, I, R, V, T, P, and K) was selected as variable modifications. Five amino acids including M, C, W, Y, and F, were selected for double oxidation variable modification. Identified peptides with their corresponding proteins were exported as an excel file together with an MzIdentML file, which were used for protein quantitation in Skyline software.[5] Following data analysis of the extents of oxidation at site-specific and protein levels were conducted with R Studio with customized R scripts. The raw data and corresponding data searching results were uploaded to Pride database.

Determination of AAPL Values. Extent of interactions reveal by oxidation was determined using the equation:

$$AAPL = Q_{Protein} \times log\frac{Q_{Protein}}{E_{Protein}}$$

where $Q_{protein}$ and $E_{protein}$ were defined as follows.

$$Q_{Protein} = \frac{EPO_{M}}{EPO_{Ctrl}} \times Spectral\ Count_{M}$$

$$E_{Protein} = \frac{Row\ Sum_{Protein} \times Column\ Sum_{Condition}}{Table\ Sum}$$

Here, EPO is the extent of protein oxidation, and it is defined as the following equation.

$$EPO = \frac{\sum Intensity_{oxidized\ peptides}}{\sum Intensity_{all\ peptides}}$$

The equation was modified from analogous interactions using a protein-pull down procedure of a target protein and its interacting partners by Sardiu *et al.*[6]. In those experiments, a protein was tagged and pulled down with activated beads using biotin-avidin technology. In the AAPL method the relationships between the target proteins and its interacting partners were determined based on the EPO combining with the spectral count of the oxidized protein.

SUPPLEMENTARY INFORMATION

Generation of antibody probes

The glycan modification was first performed on an IgG commercial protein standard and then on selected antibodies. To determine the extent of the azido modification, the glycans were released with PNGase F enzyme and subjected to nanoLC-MS/MS characterization. The total ion

chromatograms (TIC) of the released glycans from both modified and native IgG proteins are shown in **Figure S1(a)** and **(b)**. The unmodified IgG yielded the expected N-glycan profile with no azido-modified compounds. In the glycan-modified IgG, a number of azido-containing compounds were observed in the total ion chromatogram (TIC - peaks in dark blue). Further quantitation revealed that after modification, the GalNAz-containing compounds accounted for 50% of the total glycan abundances. Additionally, an alternative approach was employed to produce the antibody probe where the FeBABE was directly added through the reaction of the FeBABE reagent with free sulfhydryl groups on cysteines of the polypeptide backbone. The resulting antibody product contained the FeBABE probe as part of the polypeptide backbone (**FeAb**). The antibodies were not saturated with either type of probes (FeDBAb and FeAb). We examined the effect of saturation by increasing the amount of FeDBAb and FeAb substrates relative to the amount of starting antibody material (**Table S1**). The resulting antibody probes yielded varying amounts of oxidation (See main context).

To further examine the binding properties of the modified antibodies (**FeDBAb** and **FeAb**), immunofluorescence with confocal microscopy was conducted. Cells were treated with natural and modified antibodies and stained for plasma membrane (green color) and nuclear (blue color) for confocal imaging. For the validation of modified anti-ERBB2 antibody, the SKBR3 cell line with ERBB2 protein highly expressed was selected for imaging, which is known as a standard in vitro model for breast cancer.[7] The imaging of surface ERBB2 using original anti-ERBB2 antibody, modified anti-ERBB2-FeAb, and anti-ERBB2-FeDBAb are demonstrated in **Figure S2a-c**, respectively, and it was observed that the cell membrane was colocalized with both original and modified antibody probes. The staining pattern of the cell membrane with CellMask Orange stain was homogeneous throughout the membrane, while the antibody stain clustered in parts.

SUPPLEMENTARY FIGURES

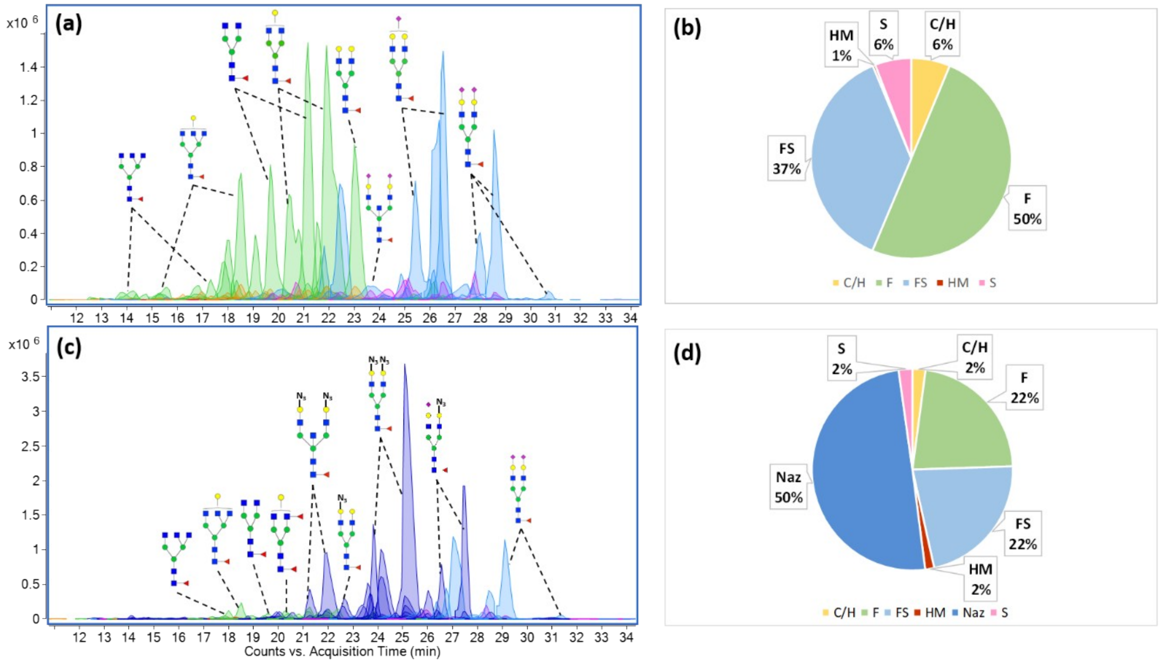

Figure S1. The modification of antibody glycans with GalNAz. a. The glycomic profile of unmodified IgG. Peaks of different types of glycans are color coded and tentative structures of N-glycans are assigned to peaks. **b.** The composition of different types of glycans. **c.** The glycomic profile of IgG modified with GalNAz. Peaks of GalNAz modified glycans are in dark purple. **d.** The composition of different types of glycans.

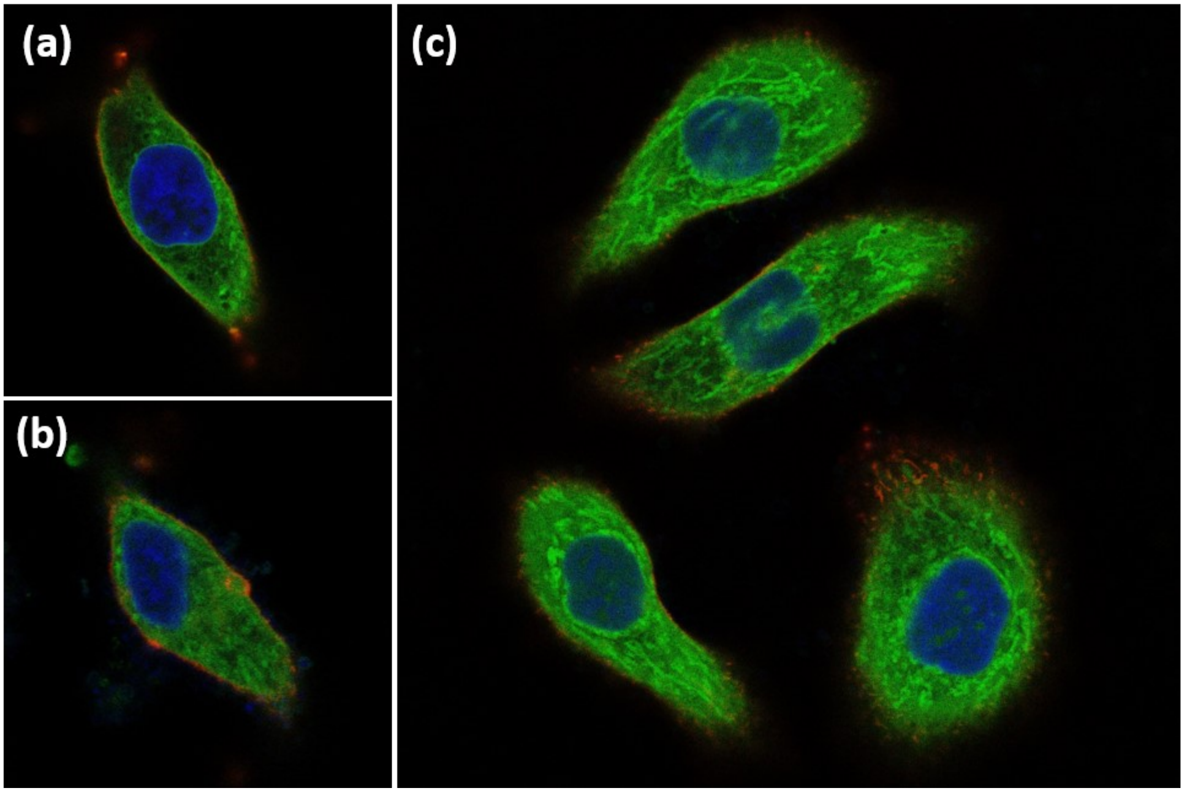

Figure S2. The immunofluorescence of SKBR3 cells using anti-ERBB2 antibody. a. The confocal imaging of ERBB2 with non-modified anti-ERBB2 antibody. **b.** The confocal imaging of ERBB2 with anti-ERBB2-FeAb. **c.** The confocal imaging of ERBB2 with anti-ERBB2-FeDBAb. (Color legend, red: CellMask^{TM} Deep Red Stain for plasma membrane; blue: Hoechst 33342 for nuclei; green: Alexa Fluor 488 anti-ERBB2 antibody.

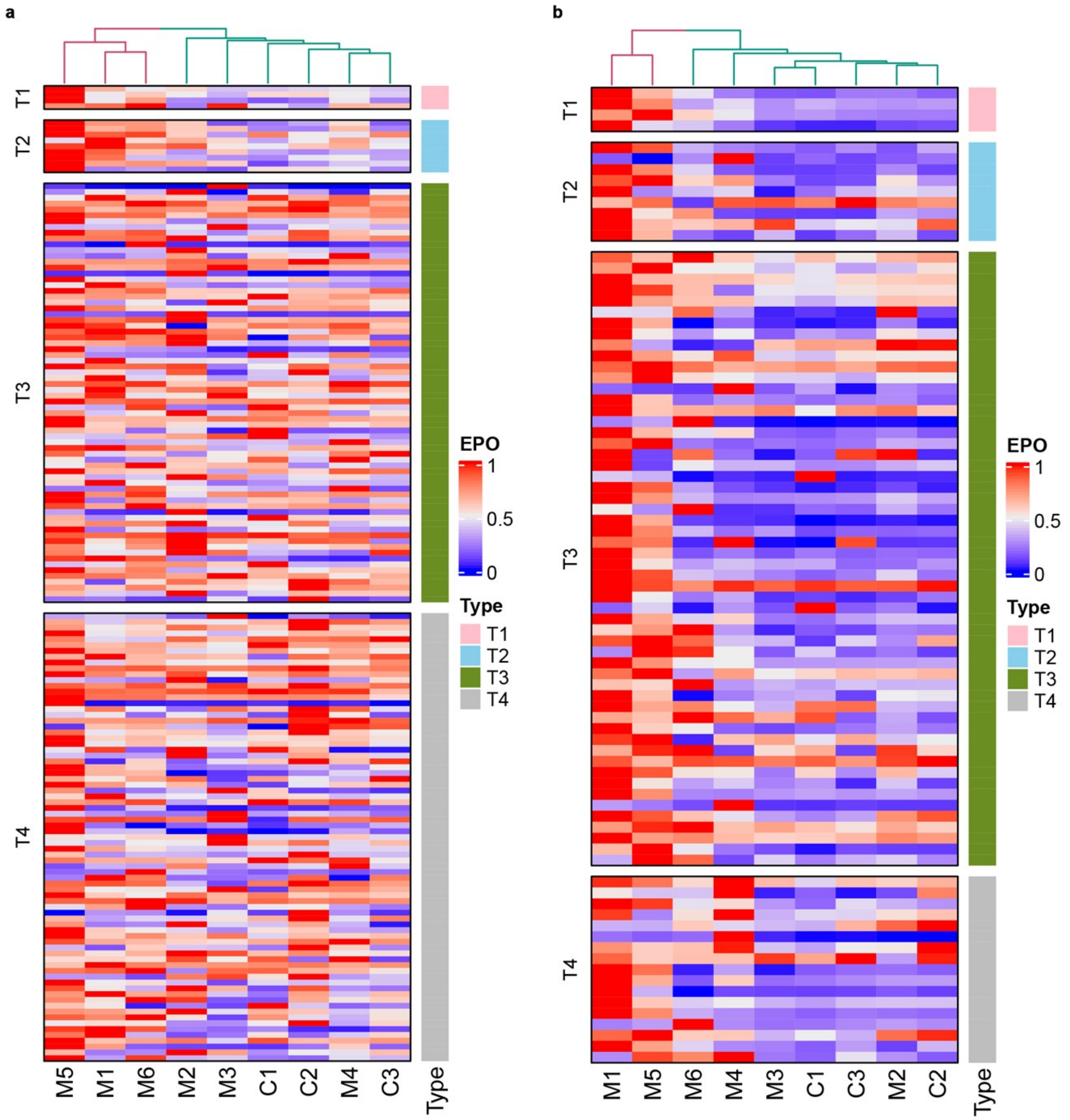

Figure S3. The heatmaps of protein extent of oxidation under different conditions for AAPL method optimization in a. PNT2 cells with modified anti-ATP1A1 antibody and **b.** SKBR3 cells with modified anti-ERBB2 antibody. Proteins are clustered based on their STRING types. Methods giving similar oxidation patterns across all the oxidized proteins are clustered together, using the hierarchical clustering algorithm in R

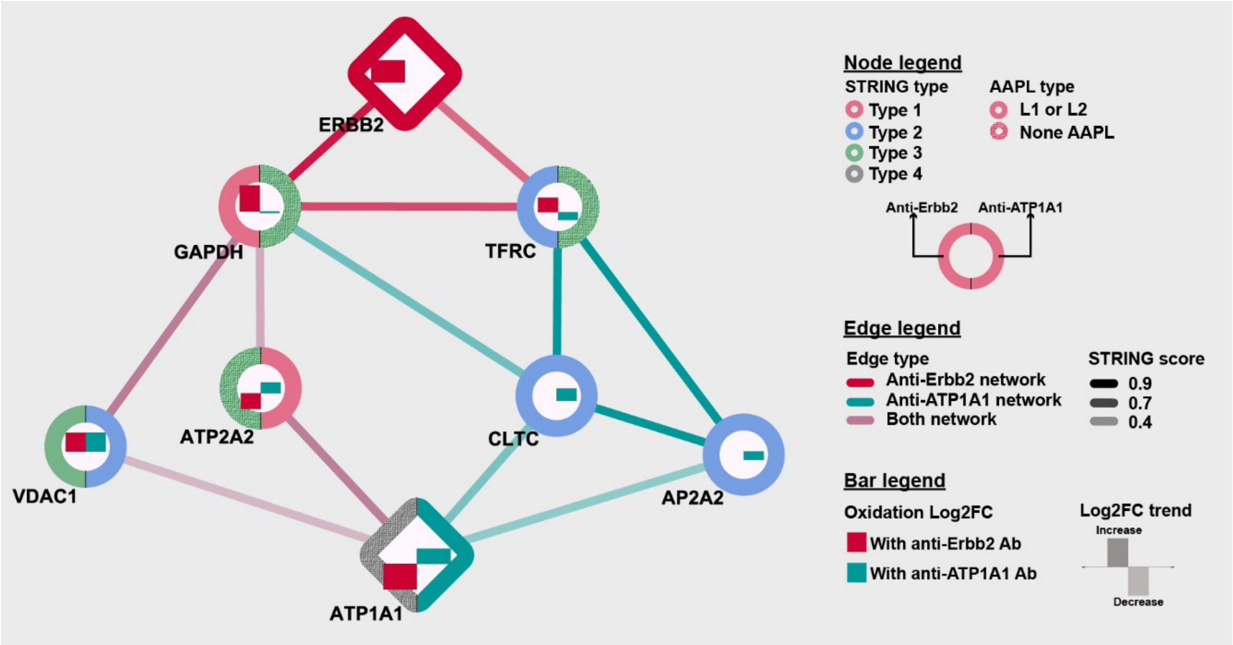

Figure S4. Merged interaction network of ATP1A1 and ERBB2. To demonstrate the STRING connection of GAPDH and TFRC with ATP1A1 in ATP1A1 network, two more interactors of ATP1A1 including CLTC and AP2A2 were also included in the merged network. In the merged network, the node edge color represents the STRING types of each protein in ATP1A1 network (right semi-circle) and ERBB2 network (left semi-circle). For identified AAPL L1 or L2 interactor, its node edge is represented with pattern, while node edge of non AAPL interactor is represented without pattern. Inside of each node, the EPOs of each protein in both networks were also illustrated as a bar graph.

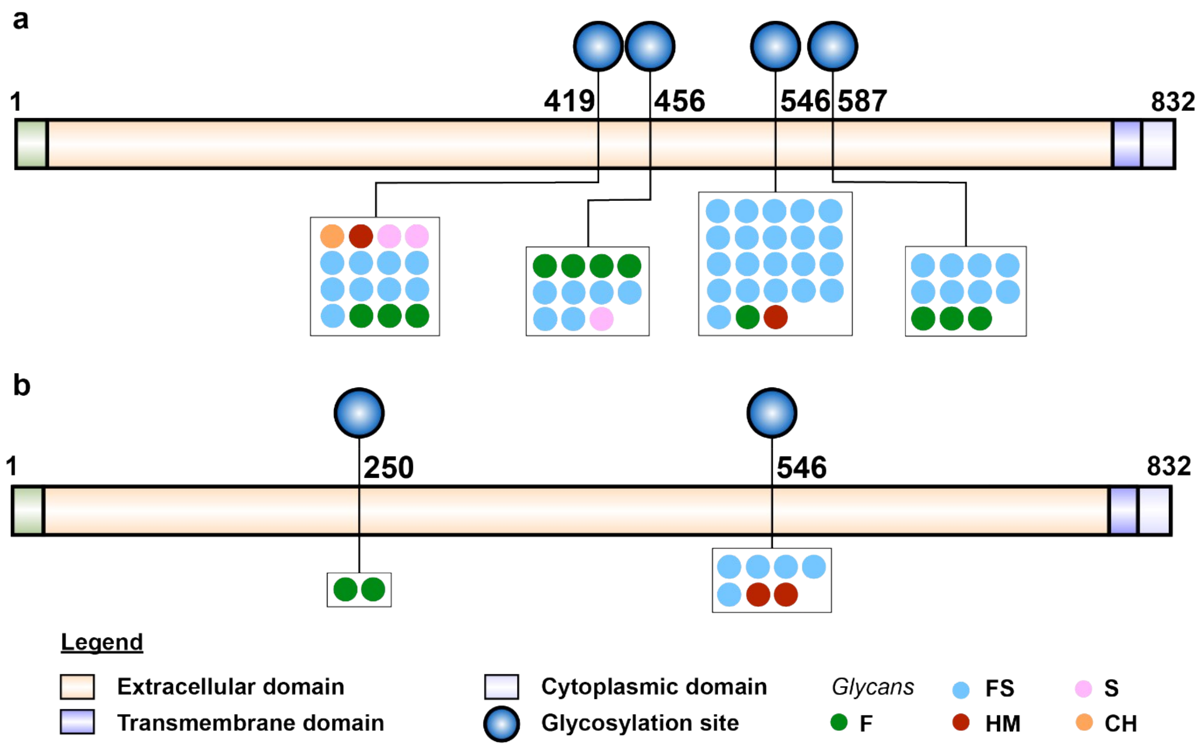

Figure S5. Site specific glycosylation of LI-cadherin in **a.** differentiated Caco-2 cells and **b.** undifferentiated Caco-2 cells. Glycosylation sites are represented with blue circles and labeled with site positions. The abundance of each type of glycan modification is demonstrated as the number of colored circles beneath each site, representing the spectral count of each type of glycosylation obtained from glycoproteomics results.

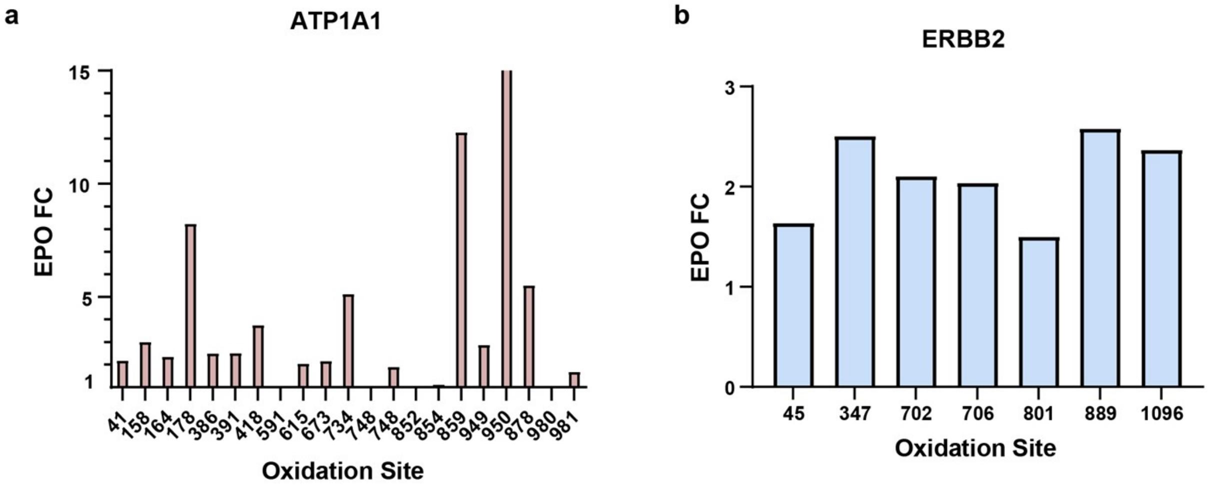

Figure S6. Site specific extent of protein oxidation (EPO) of a. ATP1A1 and **b.** ERBB2. The fold change (FC) of EPO is generated with the M5 against the control. For ATP1A1, sites 878, 980, and 981 are located extracellular membrane domains. Both sites 878 and 981 are having EPO FC values larger than 1.5. For ERBB2, sites 45 and 347 are located extracellular membrane domain, and their EPO FC values are above 1.5.

SUPPLEMENTARY TABLE

Conditions	Type of Ab	Type of Fe(III) probe	Amount of Ab	Ratio of Ab:Fe(III)
C1	/	/	/	/
C2	Unmodified Ab	/	10 µg	/
C3 (H_2O_2 only)	/	/	/	/
M1			10 µg	2:1
M2	FeAb	FeBABE	10 µg	1:1
M3			10 µg	1:2
M4			10 µg	1:5
M5	FeDBAb	DBCO-FeBABE	20 µg	1:5
M6			30 µg	1:5

Supplementary Table 1. The details of reaction and control conditions of AAPL method.

REFERENCES

1. Li, Q., Xie, Y., Xu, G. & Lebrilla, C. B. Identification of potential sialic acid binding proteins on cell membrane by proximity chemical labeling. *Chemical Science* (2019).
2. Owens, J. T. *et al.* Mapping the σ⁷⁰ subunit contact sites on Escherichia coli RNA polymerase with a σ⁷⁰-conjugated chemical protease. *Proceedings of the National Academy of Sciences* **95**, 6021-6026, doi:10.1073/pnas.95.11.6021 (1998).
3. Liu, H. & May, K. Disulfide bond structures of IgG molecules. *mAbs* **4**, 17-23, doi:10.4161/mabs.4.1.18347 (2012).
4. Li, Q., Xie, Y., Wong, M., Barboza, M. & Lebrilla, C. B. Comprehensive structural glycomic characterization of the glycocalyxes of cells and tissues. *Nature Protocols* **15**, 2668-2704 (2020).
5. MacLean, B. *et al.* Skyline: an open source document editor for creating and analyzing targeted proteomics experiments. *Bioinformatics* **26**, 966-968 (2010).
6. Sardiu, M. E. *et al.* Topological scoring of protein interaction networks. *Nature communications* **10**, 1-14 (2019).
7. Holliday, D. L. & Speirs, V. Choosing the right cell line for breast cancer research. *Breast cancer research* **13**, 215 (2011).
